# PET/CT imaging of esophageal cancer targeting tumor cell specific αvβ6-integrin expression

**DOI:** 10.1007/s00259-025-07408-7

**Published:** 2025-06-20

**Authors:** Kateřina Dvořáková Bendová, Tanja Groll, Barbora Neužilová, Kristýna Krasulová, Zbyněk Nový, Falco Reissig, Katja Steiger, Melanie Boxberg, Elisabeth Eppard, Jan Wuestemann, Marián Hajdúch, Moritz Jesinghaus, Jakub Šimeček, Michael C. Kreissl, Miloš Petřík, Johannes Notni

**Affiliations:** 1 Institute of Molecular and Translational Medicine, Faculty of Medicine and Dentistry, Palacký University Olomouc, Olomouc, Czech Republic; 2 Institute of Pathology, School of Medicine and Health, Technical University of Munich, Munich, Germany; 3 Czech Advanced Technology and Research Institute, Palacký University Olomouc, Olomouc, Czech Republic; 4 TRIMT GmbH, Carl-Eschebach-Str. 7, 01454 Radeberg, Germany; 5 Division of Nuclear Medicine, Department of Radiology and Nuclear Medicine, University Hospital Magdeburg, Magdeburg, Germany; 6 Laboratory of Experimental Medicine, Institute of Molecular and Translational Medicine, University Hospital Olomouc, Olomouc, Czech Republic; 7 Institute of Pathology, University Hospital Marburg, 35043 Marburg, Germany

**Keywords:** Esophageal Cancer, Surveillance, Positron Emission Tomography, Integrins, Theranostics

## Abstract

**Purpose:**

To assess the potential of αvβ6-integrin as a theranostic target in esophageal cancer.

**Methods:**

Membranous β6-integrin (ITGB6) expression was analyzed in 306 specimens of human esophageal squamous cell carcinoma (ESCC) obtained by immunohistochemistry (IHC) from 100 patient cases (1, 37, 58, and 4 of grade G1, G2, G3, and G4, respectively). Ga-68 labeling of D0103 was done manually for preclinical experiments and fully automated for clinical application. Preclinical characterization of Ga-68-D0103 was performed in SCID mice bearing subcutaneous xenografts of H2009 (αvβ6-positive) or MDA-MB-231 (αvβ6-negative) carcinoma cell lines, by ex vivo biodistribution (10, 30, 90, and 180 min p.i) and PET imaging (30, 90, and 180 min p.i.)., without and with co-injection of gelofusine (4% succinylated gelatin). A patient with type-II diabetes (f, 68y, 115 kg) with proximal G2 ESCC was investigated by Ga-68-D0103 PET/CT (193 MBq) at 15, 45, 90, and 104 min p.i..

**Results:**

99% of ESCC cases were found β6-integrin positive by IHC, of which 48%, 31%, and 20% showed strong, moderate, and low ITGB6 expression, respectively, with no correlation to tumor grade. Ex vivo biodistribution of Ga-68-D0103 in H2009 xenografted mice after 30, 90, and 180 min showed tumor-to-blood ratios of 6.8, 37, and 124, respectively; tumor-to-muscle ratios of 12, 14, and 36, respectively; tumor-to-liver ratios of 10, 17, and 14, respectively; and tumor-to-pancreas ratios of 20, 47, and 56, respectively. Co-administration of gelofusine did not change the tumor uptake but reduced the kidney uptake by 89% (from 178%iA/g to 19.1%iA/g, 90 min p.i.), resulting in an 8.7-fold higher tumor/kidney ratio. µPET imaging in H2009 xenografted mice confirmed a high tumor uptake and low background already 30 min p.i.. Blockade biodistribution and µPET in αvβ6-(–) MDA-MB-231 mice demonstrated target specificity. Clinical PET/CT of a patient with ESCC showed increasing tracer uptake over time in the primary tumor (SUVmax 9.0 and 11.3 at 15 and 104 min p.i., respectively) and in a lymph node metastasis (SUVmax 19.5 and 28.3, respectively), and a decreasing blood pool activity (SUVmean 2.75 and 0.98, respectively).

**Conclusions:**

High (99%) membranous expression frequency and density on tumor cells underscores the potential of αvβ6-integrin as a theranostic target in ESCC, suggesting that αvβ6-integrin PET/CT imaging may adopt a role in re-staging and therapy guidance in this cancer type. The prolonged tumor retention furthermore indicates a therapeutic potential of αvβ6-integrin targeted radiopharmaceuticals when labeled with radionuclides such as lutetium-177, terbium-161, or actinium-225.

**Supplementary Information:**

The online version contains supplementary material available at 10.1007/s00259-025-07408-7.

## Introduction

Integrins are a class of 24 transmembrane cell surface receptors, many of which are involved in cancer development and progression [1, 2]. αvβ6-Integrin is frequently overexpressed by carcinoma cells and is the likely most important activator of TGF-β [3]. Latent (inactive) TGF-β is secreted by virtually all mammalian cells into the extracellular space as a protein complex with latency-associated peptide (LAP), which, in turn, is bound to the extracellular matrix as the large latent complex (LLC). Activation of TGF-β starts with binding of αvβ6-integrin to an RGD-recognition motif in LAP, whereafter a force is transmitted from the intracellular actin cytoskeleton via the β6 subunit. The exerted force leads to deformation of LAP, thus releasing TGF-β in its freely diffusible form capable of binding to its receptors for signaling [4, 5]. TGF-β generally acts as a growth inhibitor in normal tissues and early stage cancer [6]. However, once the tumor cells have become insensitive to the anti-growth signals of TGF-β [7] by a loss of downstream components of the TGF-β signaling pathway, such as p53 [8] or Smad4 [9], TGF-β promotes infiltrative growth and tumor malignancy by augmenting cellular transformation, epithelial-mesenchymal-transition driven invasion, metastasis [10], and particularly mediates suppression of the antitumor immune reaction by inhibiting cytotoxic T-cells and natural killer (NK) cells [11]. As these pro-oncogenic roles of TGF-β becomes particularly relevant in late-stage cancer, the expression of its main activator αvβ6-integrin is also linked to malignancy. Hence, αvβ6-integrin is upregulated in various malignant cancers [12], for example, in pancreatic ductal adenocarcinoma (PDAC) [13], oral squamous cell carcinoma (OSCC) [14], ovarian [15] and cervical cancer [16], and in non-small cell lung cancer (NSCLC) [17] and its brain metastases [18].

**Fig. 1:**
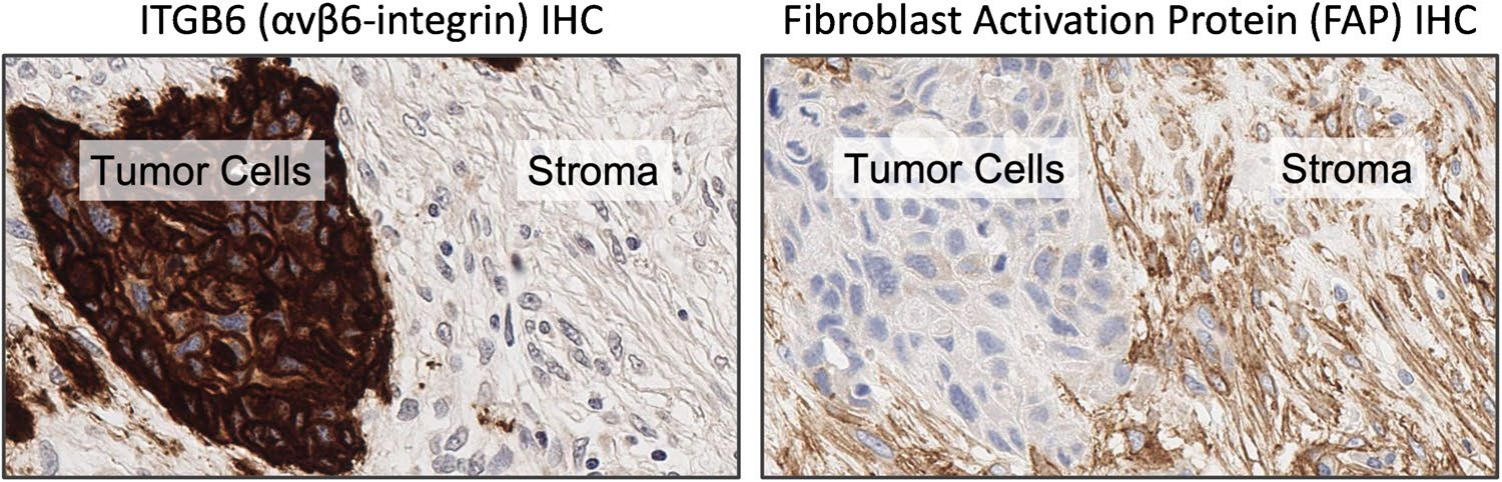
Exemplary ITGB6 and fibroblast-activation protein (FAP) IHC in adjacent tissue sections of esophageal squamous cell carcinoma (ESCC). Tumor cells show strong membranous αvβ6-integrin and no FAP expression, whereas stromal fibroblasts typically express FAP but no αvβ6-integrin

As a cell adhesion protein, αvβ6-integrin is typically found on tumor cell membranes. After binding to peptidic ligands, the ligand-receptor complex is internalized within 30–60 min [19]. This mechanism can be exploited for targeted drug delivery [20], e.g., to selectively kill tumor cells in vivo with peptide-drug conjugates comprising cytotoxic drugs and αvβ6-integrin binding peptides [21]. The ability to selectively deliver payloads (such as radionuclides, cytostatics, or siRNA) into the cytoplasm of tumor cells is perhaps the most important difference to another popular theranostic target, fibroblast activation protein (FAP) [22, 23]. FAP is not normally expressed by tumor cells but by mesenchymal cells of many solid carcinomas [24], especially cancer-associated fibroblasts (CAFs) [25], which are typically αvβ6-integrin-negative in esophageal squamous cell carcinoma (ESCC; see an example in Fig. 1).

Taken together, αvβ6-integrin is a valuable target for imaging, not only in the context of theranostic radioligand pairs but also for image-based patient selection for αvβ6-integrin targeted therapies [21], including but not limited to antibody–drug conjugates for anticancer treatment [26, 27]. Against this background, various αvβ6-integrin targeted PET radiopharmaceuticals have been developed and applied in cancer patients [28–36]. To date, the largest number of clinical αvβ6-integrin PET/CT applications in oncology have been reported for the peptide trimer ^68^Ga-Trivehexin, for example, in PDAC [37, 38], head-and-neck squamous cell carcinoma (HNSCC) [39, 40] and its brain metastases [41], non-small-cell lung cancer (NSCLC) [42], parathyroid adenoma [43] and carcinoma [44], bronchial mucoepidermoid carcinoma [45], papillary thyroid carcinoma [46], and lobular [47] as well as lymphatically metastasized breast cancer [48].

The current work elucidates the clinical relevance of targeting αvβ6-integrin in the context of ESCC. 306 human ESCC tissue specimens were analyzed by immunohistochemistry (IHC) for membranous expression density and frequency of β6-integrin (ITGB6), which only dimerizes with αv-integrin and thus is limiting and indicative for actual membranous αvβ6-integrin abundance [13]. We furthermore characterized and evaluated the αvβ6-integrin targeted PET tracer ^68^Ga-D0103 for αvβ6-integrin targeted PET/CT imaging of locally advanced and metastatic esophageal cancer.

## Materials and methods

### Immunohistochemistry

A tissue microarray (TMA) cohort consisting of 100 human esophagus cancer cases of the squamous cell carcinoma type (ESCC) was evaluated. Each case was represented by 1–8 TMA cores (306 cores analyzed in total). Tissue samples were fixed in 10% neutral-buffered formalin and routinely processed for histology. β6-integrin (ITGB6) staining was performed on a Leica Bond Rx^m^ autostainer (Leica Biosystems, Wetzlar) using an anti-human β6-integrin antibody [clone 442.5C4] (#407317, dilution 1:100, Merck Millipore, Burlington, Massachusetts, USA); antigen retrieval with enzyme pretreatment (Bond™ Enzyme Pretreatment E1) for 5 min (#AR9551, Leica Biosystems, Wetzlar, Germany); visualization of antibody binding with brown chromogen (3,3’-diaminobenzidine (DAB) (#DS9800, Bond Polymer Refine Detection, Leica Biosystems, Wetzlar, Germany). Slides were digitized (Aperio AT2, Leica) and evaluated using a digital microscopy software (Aperio ImageScope, Leica). All cores (*n* = 306) were individually evaluated regarding immunoreactivity for β6-integrin in terms of a positive signal (brown DAB precipitate) of the tumor cell membranes. IHC readout was done according to Sipos et al. [49]. Membranous staining intensity was assessed using a 4-level scoring scheme (0: no membranous staining, 1, 2, and 3: low, moderate, and strong membranous staining, respectively), referring to the membranous staining intensity of the majority of the tumor cells in a given specimen and disregarding any cytoplasmic staining. In addition, the percentage (frequency) of β6-integrin positive tumor cell membranes was assessed for each tumor core. To determine a final case score, the β6-integrin staining intensity of the majority of tumor cells per core was multiplied with the frequency of membranous positive tumor cells per core, and individual core scores were averaged if the number of cores was > 1 for a given case. Case scores were categorized into the following ITGB6 expression levels: negative (**0**), score 0; low (**1**), scores > 0–1; moderate (**2**), score > 1–2; strong (**3**), score > 2–3.

FAP IHC (Fig. 1) was done with anti-human FAP monoclonal rabbit antibody [clone EPR20021] (abcam, # ab207178) diluted 1:100, with a 30 min heat pretreatment using EDTA based pH 9 Epitope Retrieval Solution 2 (#AR9640, Leica Biosystems, Wetzlar, Germany).

### Radiopharmaceuticals

GaCl_3_ was obtained from a ^68^Ga/^68^Ge-generator (Eckert & Ziegler Eurotope GmbH, Berlin, Germany) by a fractionated elution method using 0.1 M HCl [50]. The radiolabeling precursors Trivehexin and D0103 were synthesized according to previously published protocols [36, 51, 52]. ^68^Ga-radiolabeling was done by mixing a solution of sodium acetate (30 µL, 155 mg/mL in water) and 300 µL of generator eluate (80–85 MBq) with either 10 µg (for in vivo imaging) or 25 µg (for ex vivo biodistribution studies) of D0103 or Trivehexin. The mixture was incubated at 95 °C for 15 min. Sodium acetate (100 µL) was then added to raise the pH to ~ 6.

The radiochemical purity of the radiotracers was evaluated immediately after radiolabeling using radio-RP-HPLC, consisting of a Dionex UltiMate 3000 (Thermo Scientific, Waltham, Massachusetts, USA) with a GABI Star radiation detector (Raytest, Straubenhardt, Germany); column: Nucleosil 120–5 C18 250 × 4 mm (WATREX, Prague, Czech Republic); flow rate: 1 mL/min; oven temperature: 25 °C; UV detection wavelengths 225 nm and 250 nm; mobile phase gradient: acetonitrile (ACN) with 0.1% trifluoroacetic acid (TFA) in water; 0–3 min, 0% ACN; 3–6 min, 0–50% ACN; 6–10 min, 50–80% ACN; 10–13 min, 80% ACN; 13–15 min, 0% ACN.

### Cell culture

The human lung adenocarcinoma cell line NCI-H2009 (ATCC, Virginia, USA) was cultured in Dulbecco's modified Eagle's medium (D-MEM) supplemented with 10% fetal bovine serum. Human mammary gland adenocarcinoma cell line MDA-MB-231 (ATCC, Virginia, USA) was cultured in RPMI-1640 medium supplemented with 10% fetal calf serum. Media supplements were purchased from Merck (Darmstadt, Germany). Cells were incubated at 37 °C in a 5% CO_2_ humidified incubator and subcultured at a confluence of 70–90%. Cell number and viability were determined using the Vi-CELL™ Cell Viability Analyzer (Beckman Coulter, California, USA). Expression of αvβ6-integrin in H2009 xenografts, and complete absence of αvβ6-integrin in MDA-MB-231 xenografts, has been demonstrated previously [39].

### Animal experiments

All animal experiments were approved by the Czech Ministry of Education, Youth, and Sports (MSMT-35035/2019–3) and the Institutional Animal Welfare Committee of the Faculty of Medicine and Dentistry, Palacký University, Olomouc, and were performed in accordance with the regulations and guidelines of the Czech Animal Protection Act (no. 246/1992). Female 6–8 week old SCID mice (Envigo, Horst, The Netherlands) were used for animal experiments in this study. The animals were acclimatized to the laboratory conditions for at least one week prior to the experiments. Animals were housed in groups of 5–6 in individually ventilated cages on sawdust with free access to animal chew and water. The general health of animals was monitored daily. Multiplicity of PET and biodistribution studies ranged from 4 to 5 (3 for blockade biodistribution). To avoid animal suffering and to reduce movement artifacts, retro-orbital applications and imaging were performed under 2% isoflurane anesthesia (FORANE, Abott Laboratories, Illinois, USA).

Biodistribution studies were done using tumor-bearing mice. The mice were subcutaneously injected into the right flank with 5 × 10^6^ H2009 or 2 × 10^6^ MDA-MB-231 cells, without (H2009) or combined with (MDA-MB-231) Matrigel Matrix (Corning, New York, USA) at a 1:1 ratio before application. The tumor growth was periodically monitored by caliper-based measurements. When the tumor volume reached approximately 500 mm^3^_,_ mice were used for ex vivo biodistribution or in vivo imaging studies.

For ex vivo biodistribution studies, the radiotracers prepared as described above were diluted with saline to the total volume of 2 mL. To evaluate the specificity of the uptake in tumor, a group of mice (n = 3) was pretreated r.o. with cold unlabeled D0103 10 min before the application of the radiolabeled tracer. To assess the effect of plasma expanders on the biodistribution of ^68^Ga-D0103, another group of mice (n = 4) received an i.v. injection of 100 µl of 4% succinylated gelatin (gelofusine) 5 min before application of the radiotracer. The other experimental groups were not pretreated before tracer administration. Animals received 100 µl of diluted radiotracer r.o. at an activity of ~ 3 MBq corresponding to ~ 1.25 µg (0.25 nmol) of radiotracer. The mice were sacrificed by cervical dislocation at 10, 30, 90, and 180 min post-injection. The organs of interest (blood, heart, lung, liver, spleen, pancreas, empty stomach, empty small intestine, empty large intestine, muscle, tumor, and kidneys) were collected, weighed, and the activity measured on an automatic gamma counter (2480 Wizard^2^, PerkinElmer, Massachusetts, USA). The tracer accumulation was expressed as a percentage of injected activity per gram tissue (%iA/g).

For µPET/CT imaging, animals under inhalation anesthesia were r.o. injected with 100–150 µl of radiotracers corresponding to 15–20 MBq and ~ 2.5–3.5 µg (0.5–0.8 nmol) of radiotracer per animal. All animals were placed in prone position in the Mediso NanoScan® PET/CT small animal imaging system (Mediso Medical Imaging Systems, Budapest, Hungary). Static imaging was initiated 30, 90, and 180 min post-injection. A single FOV (98.5 mm) PET lasting 15 min was performed, immediately followed by a whole-body helical CT scan (50 kVp/980 μA, 720 projections). The images were reconstructed using Mediso Tera-Tomo™ 3D PET iterative reconstruction (Mediso Medical Imaging Systems, Budapest, Hungary). Image visualization, analysis, processing and quantification were performed using Mediso InterView™ FUSION (Mediso Medical Imaging Systems, Budapest, Hungary). The scans were normalized to injected activity and animal weight.

### Clinical PET/CT imaging

The utilization of ^68^Ga-D0103 was executed on a compassionate use basis, following the acquisition of an informed consent from a 68 year-old female patient. The patient had been diagnosed with a moderately differentiated (G2) non-cornifying squamous cell carcinoma; with a tumor-node-metastasis (TPS) of less than 1%, a combined positive score (CPS) of 2, IC 2%, positive p40 staining, and positive CK5/6 staining. Given the patient´s concurrent diagnosis of diabetes mellitus type II with elevated blood glucose levels, ^68^Ga-D0103 was considered the preferred diagnostic modality over [^18^F]fluorodesoxyglucose (^18^FFDG) in this particular clinical context. The ^68^Ga-D0103 intended for clinical use was produced in accordance with cGMP guidelines and provided as a sterile, filtered, and saline-diluted solution with a specific activity of 185.8 MBq/mL. Quality control was performed in accordance with the relevant European guidelines, Eu. Ph. chapters and along the lines of existing monographs for ^68^Ga-radiopharmaceuticals. PET/CT imaging was performed on a dedicated PET/CT device (Biograph mCT 64®; Siemens Healthineers, Erlangen, Germany). An activity of 193 MBq ^68^Ga-D0103 was administered intravenously, after which PET imaging data were acquired 15, 45, and 104 min p.i.. The imaging procedure covered the entire body (skull base to mid-thigh) and provided axial bed coverage of 216 mm each (Siemens TrueV R; bed overlap, 89 mm). The low-dose CT was used for attenuation correction and anatomical mapping (tube current, 50 mA; tube voltage, 120 kV; gantry rotation time, 0.5 s; pitch, 0.8). Subsequently, a PET/CT acquisition of the chest only was performed 90 min p.i. for radiotherapy planning purpose. The PET data were reconstructed using an iterative reconstruction algorithm with a Gaussian filter.

## Results

### Analysis of ITGB6 expression in ESCC

**Fig. 2:**
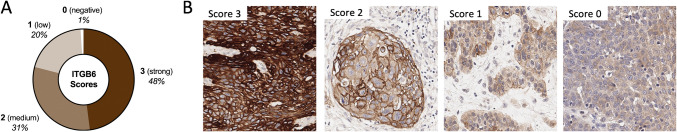
**A** Distribution of ITGB6 (β6-integrin) membranous expression levels in a 100-patient cohort of esophageal squamous cell carcinoma (ESCC). **B** Examples for immunohistochemistry (IHC) stainings for membranous ITGB6 expression scores 3, 2, and 1 (strong, moderate, and low expression, respectively) and score 0 (β6-negative). Note the diffuse cytoplasmic signal for score 0, which was not considered ITGB6-positive because only membranous ITGB6 expression was taken into account. Find more examples of ITGB6 IHC stainings in the Supplemental Information

Expression analysis was focused exclusively on membranous ITGB6 since intracellular (αv)β6-integrin cannot be directly addressed with radiolabeled peptide ligands and is therefore irrelevant in the context of PET imaging and theranostics. The final scores of 100 evaluated ESCC cases (based on 306 specimens, 1–6 per case) ranged from 0 (no membranous β6-integrin signal) to 3 (all cores per patient displayed 100% score 3). 48 (48%) of evaluated cases showed strong, 31 (31%) moderate, and 20 (20%) low positivity for avβ6-integrin (Fig. 2).

Altogether, 99 out of 100 ESCC patient cases (99%) in our cohort were found positive for membranous ITGB6 expression. Because β6-integrin only dimerizes with αv-integrin, and only the dimer αvβ6-integrin is transported to and anchored in the cell membrane [53], these figures can be considered representative for membranous αvβ6-integrin expression.

There was no correlation of tumor grade with IHC scores. In our cohort, 1, 37, 58, and 4 of the investigated cases were ESCC of grades G1, G2, G3, and G4, respectively, with IHC scores of 2.3, 1.96 ± 0.85, 1.71 ± 0.92, and 1.75 ± 1.11, respectively (averages ± SD).

### Preclinical PET tracer evaluation

**Fig. 3:**
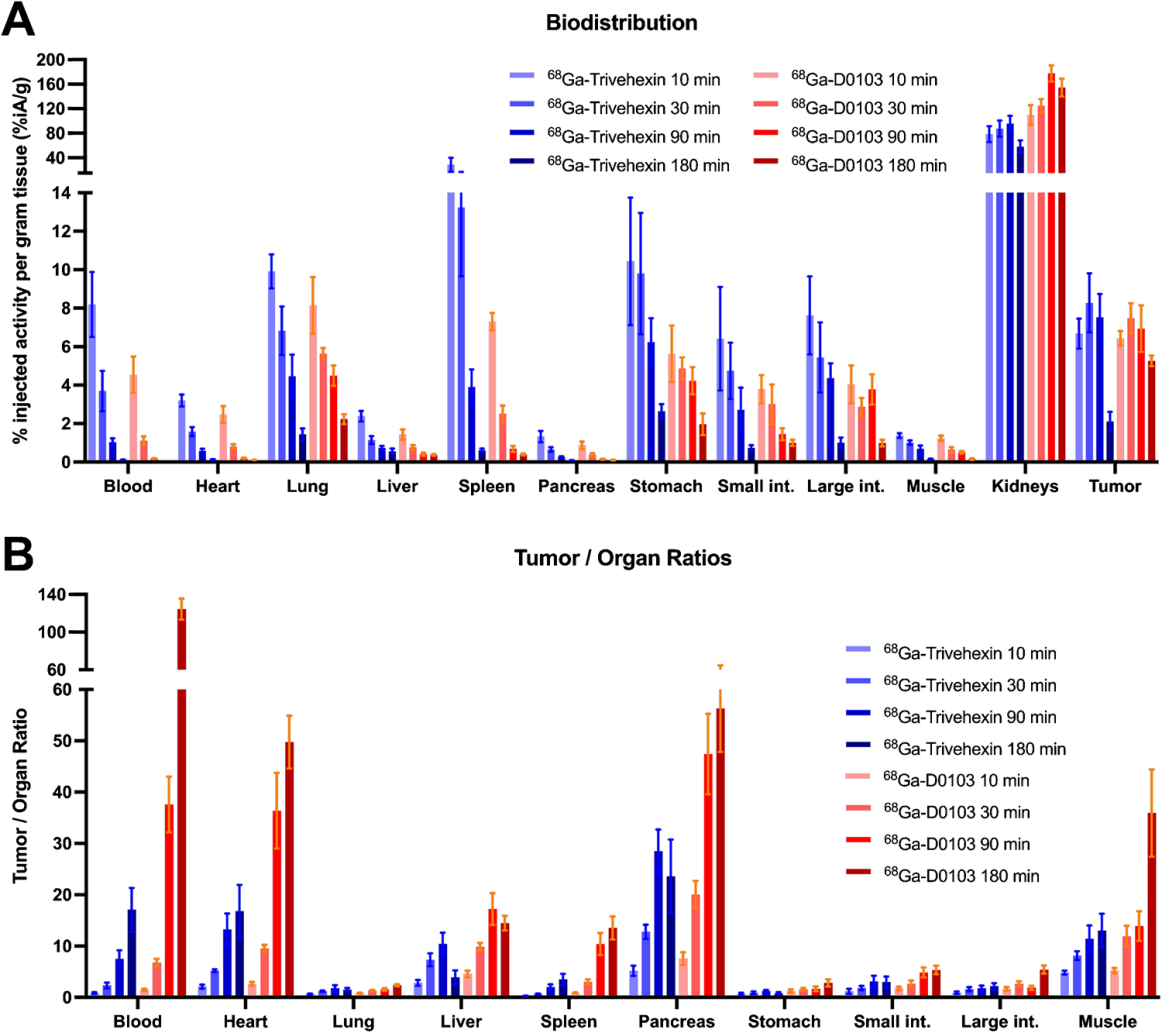
Comparison of ex vivo data for ^68^Ga-D0103 and ^68^Ga-Trivehexin, generated using mice bearing subcutaneous xenografts of H2009 (αvβ6^+^ human lung adenocarcinoma) cell lines. **A** Biodistribution at time points 10, 30, 90, and 180 min p.i. (*n* = 4–5 per group). **B** Tumor/Organ ratios at time points 10, 30, 90, and 180 min p.i

Preclinical evaluation of ^68^Ga-D0103 was done in comparison to the currently most widely used αvβ6-integrin PET radiopharmaceutical, ^68^Ga-Trivehexin. The ex vivo biodistribution profiles of ^68^Ga-D0103 and ^68^Ga-Trivehexin were investigated in severe combined immunodeficiency (SCID) mice bearing subcutaneous H2009 (human lung adenocarcinoma) xenografts up to 180 min after tracer administration. Both radiopharmaceuticals showed a comparable tumor uptake. A faster clearance from the blood pool was observed for ^68^Ga-D0103. Furthermore, a lower uptake than ^68^Ga-Trivehexin in most organs was observed, particularly in the liver, spleen, pancreas, stomach, and intestines, with exception of the kidneys (Fig. 3A). For all investigated time points (10, 30, 90, and 180 min p.i.), the tumor/blood ratios of ^68^Ga-D0103 (1.45, 6.8, 37, and 124, respectively) were markedly higher than those of ^68^Ga-Trivehexin (0.84, 2.3, 7.5, and 17, respectively), and higher tumor/organ ratios were furthermore observed for most other organs (Fig. 3B).

**Fig. 4:**
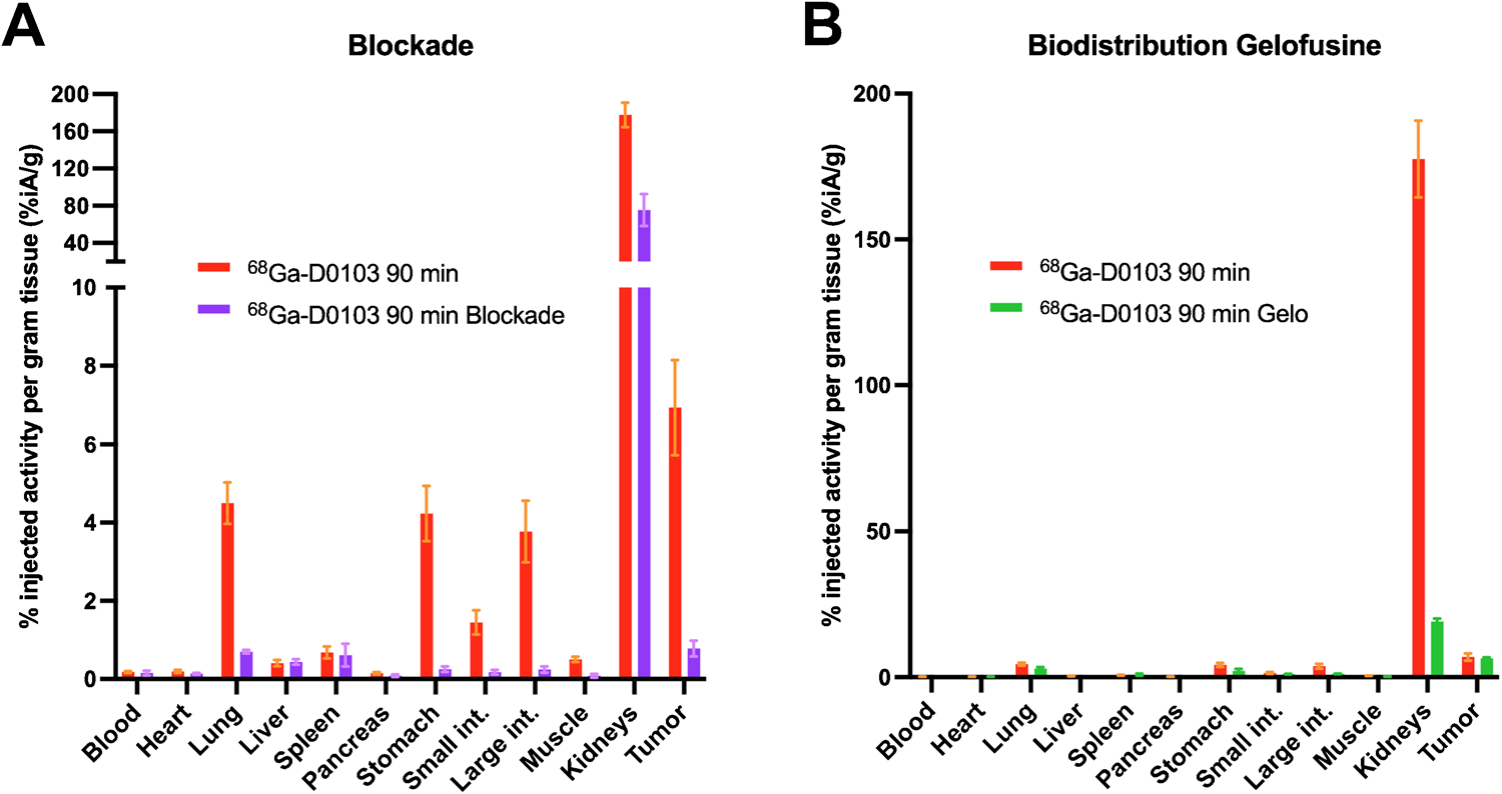
**A** Biodistribution of ^68^Ga-D0103, 90 min p.i., w/o and w/blockade (50 nmol unlabeled precursor, administered 10 min before the radioactive compound, *n* = 3). **B** Biodistribution of ^68^Ga-D0103, 90 min p.i., without and with co-injection of gelofusine (Gelo, 100 µL administered 5 min before ^68^Ga-D0103, *n* = 4), in H2009 xenografted mice

Target specificity of the tumor uptake of ^68^Ga-D0103 was confirmed by blockade with excess unlabeled precursor (50 nmol), which reduced the tumor uptake from 6.9 to 0.78%iA/g. A concomitant reduction was observed for uptake in lung, stomach, and intestines (Fig. 4A). In mice, these organs express αvβ6-integrin and thus can be considered as physiological controls [51].

To reduce renal uptake, we furthermore investigated co-injection of 4% succinylated gelatin in saline (a plasma expander, herein termed gelofusine), which was previously identified as a suitable kidney protection agent for αvβ6-integrin targeting peptide multimers [36]. Administration of 100 µL of gelofusine 5 min prior to the tracer reduced the kidney uptake of ^68^Ga-D0103 by 89% (from 178 to 19.1%iA/g, 90 min p.i.), while the tumor uptake was not significantly changed (Fig. 4B). As a result, the tumor/kidney ratio was improved from 0.039 to 0.34, i.e., by a factor of approx. 8.7.

**Fig. 5:**
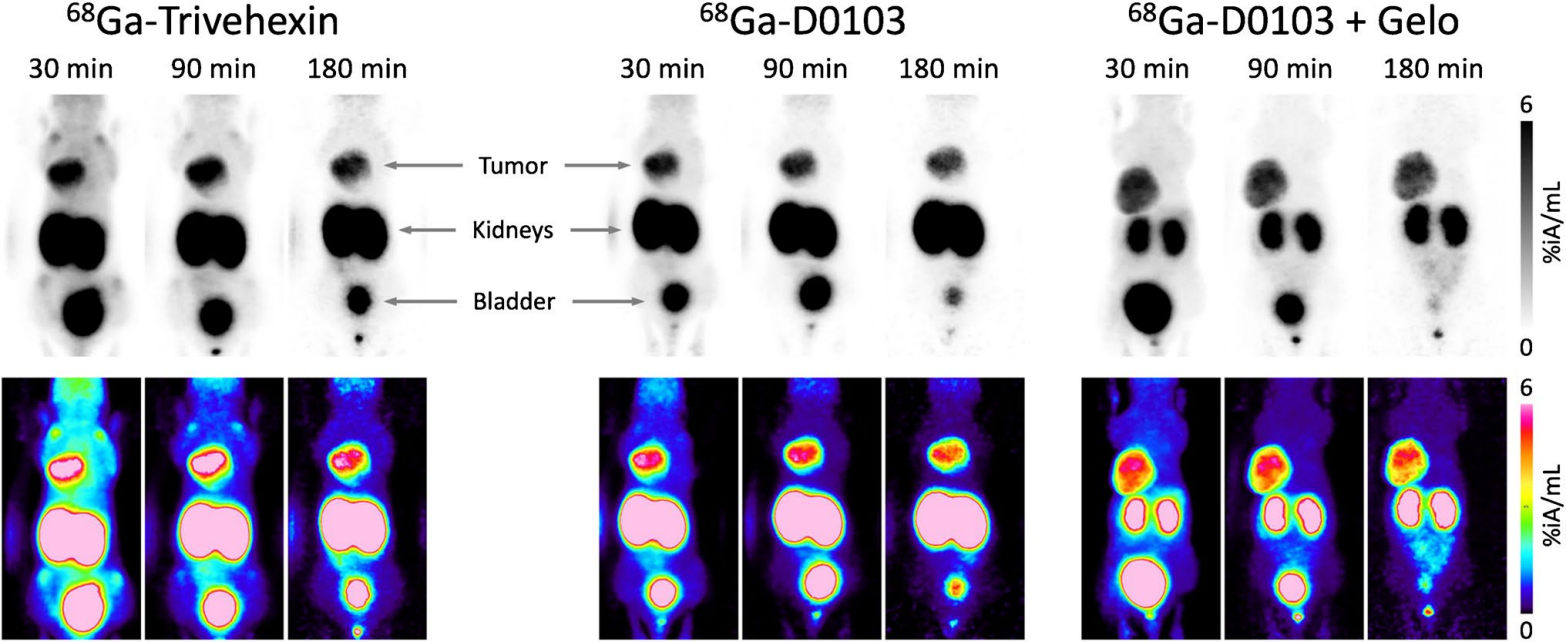
Static µPET images (maximum intensity projections) of αvβ6-integrin expressing H2009 xenografted mice, 30, 90, and 180 min p.i., recording time 15 min. Animals were imaged three times following a single injection of the respective tracer. Gelofusine (Gelo, 100 µL) was administered 5 min before ^68^Ga-D0103. The bottom row shows the same MIPs as the top row, applying a colored lookup table

**Fig. 6:**
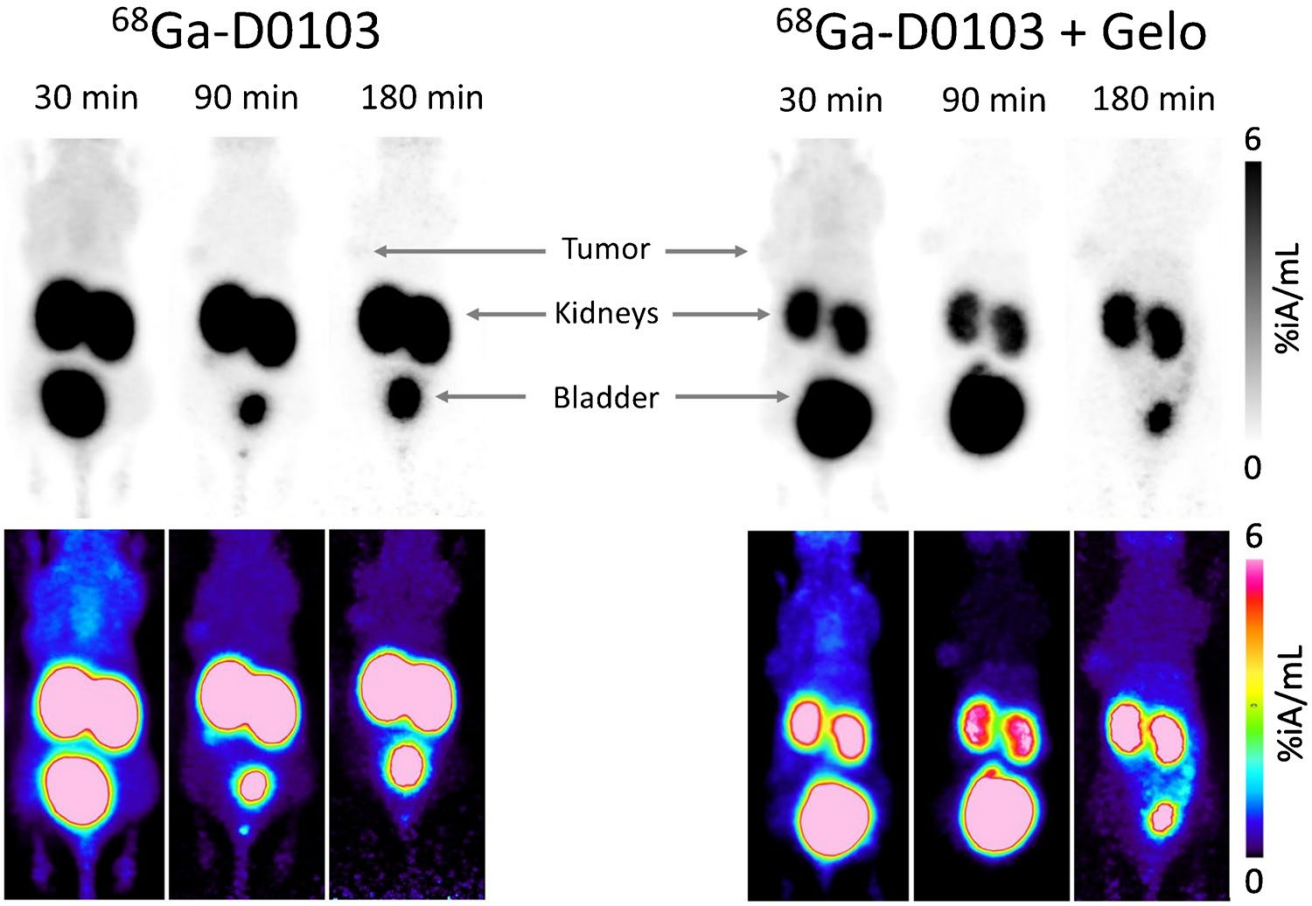
Static µPET images (maximum intensity projections, MIPs) of αvβ6-integrin negative MDA-MB-231 xenografted mice, 30, 90, and 180 min p.i., recording time 15 min. Gelofusine (Gelo, 100 µL) was administered 5 min before ^68^Ga-D0103. Animals were imaged three times following a single injection of the tracer. The bottom row shows the same MIPs as the top row, applying a colored lookup table

^68^Ga-D0103 µPET images acquired 30, 90, and 180 min p.i. in αvβ6-integrin positive H2009 mice showed a high tumor/background contrast already at early imaging time points as 30 min p.i. (Fig. 5). Pre-treatment with gelofusine, administered 5 min before ^68^Ga-D0103, resulted in a strong reduction of the kidney signal and a further reduction of the background. PET images of mice bearing αvβ6-integrin-negative MDA-MB-231 xenografts mice showed no signal in the tumors, indicating that ^68^Ga-D0103 accumulation in H2009 tumor tissue was target specific (Fig. 6).

### Clinical PET/CT imaging using ^68^Ga-D0103

**Fig. 7:**
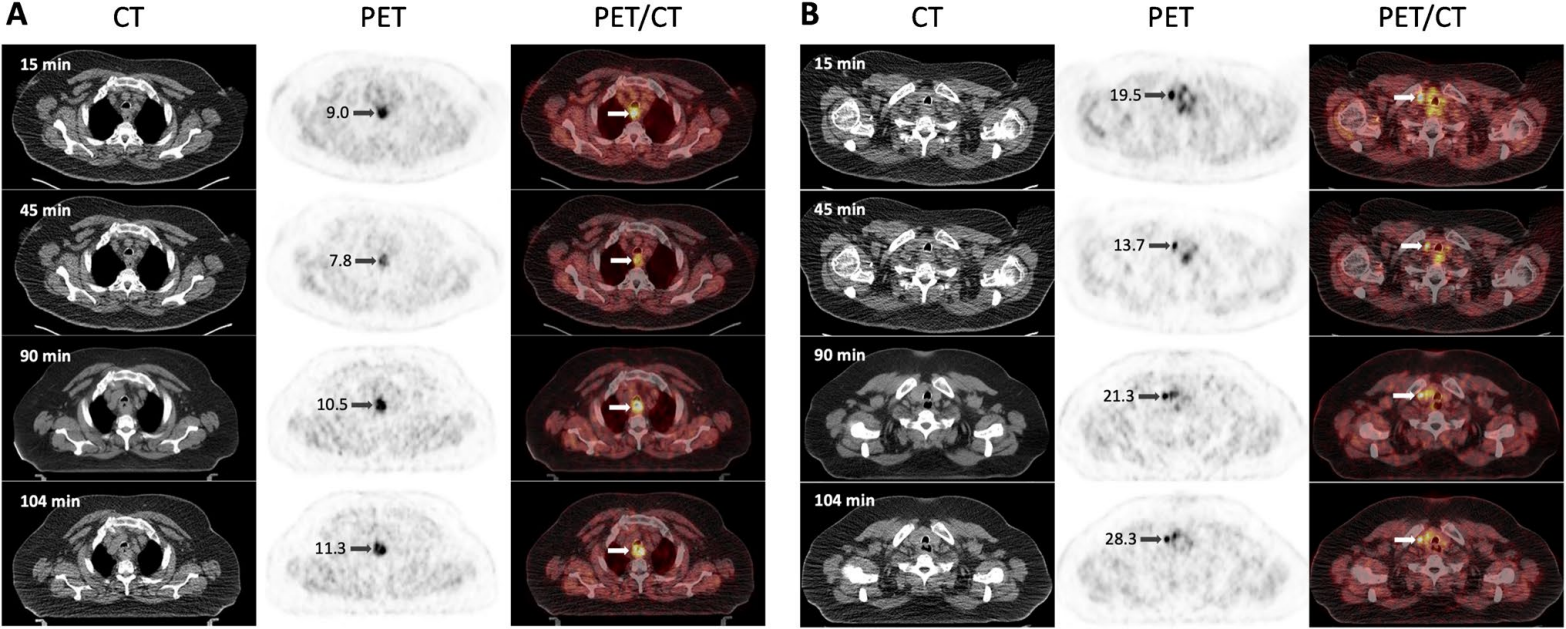
^68^Ga-D0103 PET/CT of a patient with esophageal squamous cell carcinoma (ESCC; f, 68 y, 115 kg, 193 MBq). PET is scaled to SUV 7 in all images. **A**, axial slices through primary tumor. **B**, axial slices through lymph node metastasis. Times indicate scan starts after tracer injection (p.i.). Numbers on arrows indicate the SUV_max_

Administration of ^68^Ga-D0103 was well tolerated without any adverse reactions. Rapid and increasing tracer uptake was observed in the primary tumor (SUV_max_ 9.0 and 11.3, 15 and 104 min p.i., respectively) and in a lymph node metastasis (SUV_max_ 19.5 and 28.3, respectively) (Fig. 7). The already initially low background activity further decreased over time (SUV_mean_ in the blood pool was 2.76 and 0.98; in liver, 1.84 and 1.73; 15 and 104 min p.i., respectively). Interestingly, uptake in the tumor lesions was lowest at 45 min p.i., which cannot be conclusively explained at this time due to lack of further clinical data.

**Fig. 8:**
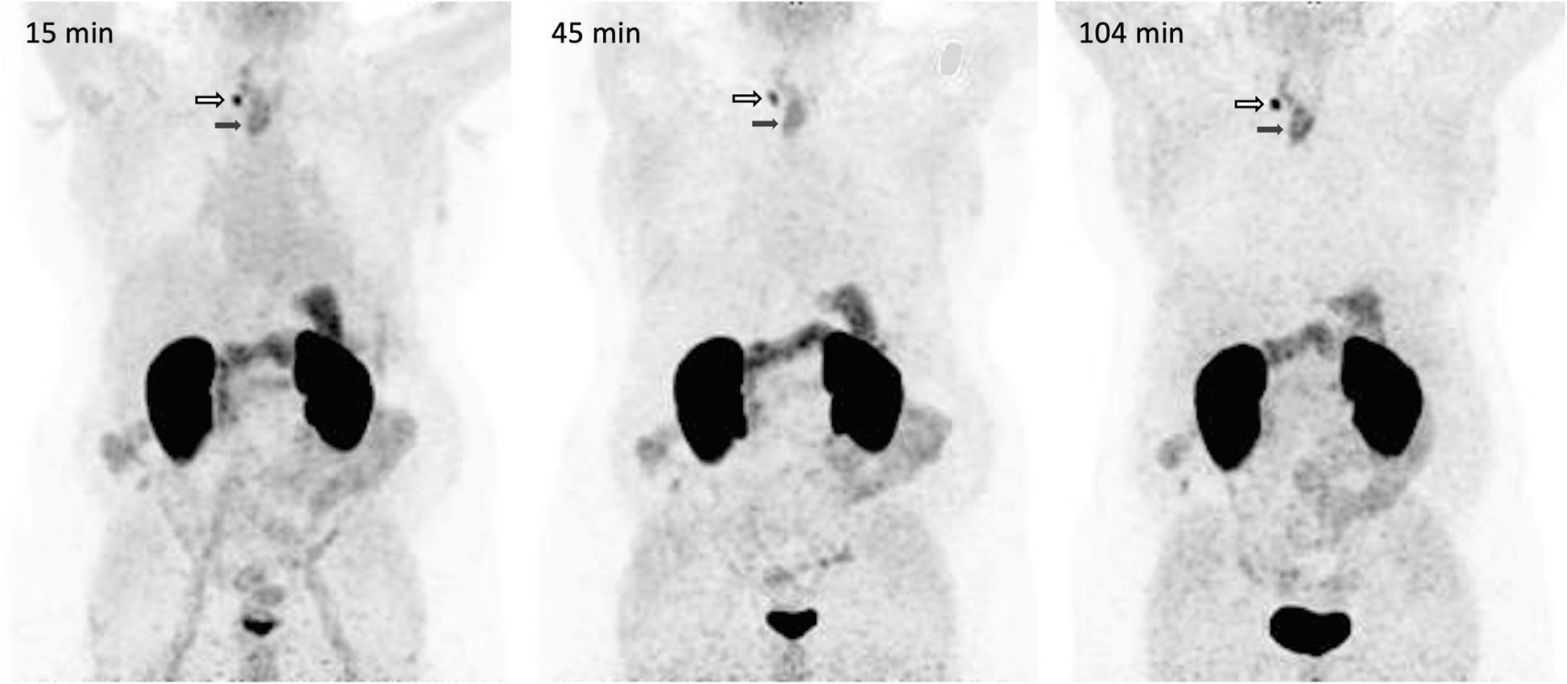
^68^Ga-D0103 PET (MIPs, scaled to SUV 15) of a patient with esophageal squamous cell carcinoma (ESCC; f, 68 y, 115 kg, 193 MBq). Times indicate scan starts after tracer injection (p.i.). Solid arrows: ESCC primary tumor. Outline arrows: lymph node metastasis

As expected from the preclinical data, a strong uptake was observed in the kidneys, which did not interfere with the observed tumor lesions (Fig. 8). In addition, a notable uptake was observed in the stomach wall, which is attributed to physiological uptake as the same phenomenon has occasionally been reported for other αvβ6-integrin targeted PET imaging agents [34, 35, 38].

## Discussion

### Imaging in ESCC management

The global incidence of esophageal cancer was about 511,000 in 2022, with an expected increase to 623,000 and 779,000 in 2030 and 2040, respectively [54]. With a low 5-year survival rate (22–24%) and a strong impact on the patients'quality of life, the disease is a rapidly growing public health problem, the main risk factors being smoking and alcohol consumption [55]. ESCC accounts for approximately 20–40% of all esophageal cancer cases in western countries, and up to 90% in the so-called “Asian esophageal cancer belt” where > 50% of all ESCC cases worldwide occur [56]. A preferred treatment strategy for locally advanced and/or lymphatically metastasized ESCC is induction or definitive RCT, followed by surgical resection [55, 57, 59]. However, esophagus surgery or even esophagectomy is associated with a high postoperative morbidity rate of 60%, a mortality rate of up to 5%, and impairs the patients'quality of life [57, 58]. Active surveillance instead of surgery can improve patient well-being, an approach that is currently investigated in, e.g., the ESOSTRATE (NCT02551458) and SANO-2 (NCT04886635) trials. Restaging after neoadjuvant therapy is a well-known challenge in ESCC management, as the radiological appearance of the tumor and the treated positive lymph nodes can be difficult to interpret owing to induced fibrosis and ulceration. The quality of life of ESCC patients with an apparent complete clinical response (cCR) after radiochemotherapy (RCT) could be significantly improved if esophageal surgery or esophagectomy is delayed or avoided and an active surveillance strategy is applied instead [59], which requires reliable methods to distinguish between patients with pathological complete response (pCR) and cCR.

^18^F-FDG PET imaging has been a cornerstone in the evaluation and management of ESCC, serving as the standard of care for staging, restaging, and monitoring of treatment response [60]. However, specificity of ^18^F-FDG PET is limited due to its uptake in inflammatory tissues, which can lead to false-positive results and obscure accurate tumor delineation [61]. Inflammation is common in ESCC patients due to factors such as esophagitis, prior interventions, or the tumor itself inducing an inflammatory response. Non-specific ^18^F-FDG uptake may therefore compromise the accuracy of locoregional imaging, making it difficult to distinguish between malignant and benign inflammatory processes. Another complicating factor is the prevalence of comorbidities like diabetes mellitus among ESCC patients, as highlighted in our clinical case. Diabetes can affect ^18^F-FDG distribution due to altered glucose metabolism, which potentially leads to decreased tracer uptake in tumors and increased background activity [62]. Patients who have undergone interventions, such as biopsy or surgery, may exhibit altered ^18^F-FDG uptake patterns due to tissue repair processes, further limiting the specificity of ^18^F-FDG PET. From a large meta-analysis (3625 pts, 56 studies), de Gouw et al. concluded that current imaging techniques used for staging after neoadjuvant therapy (CT, ^18^F-FDG PET/CT, and endoscropic ultrasound, EUS) are unfit to guide treatment decisions [57]. Another recent meta-analysis of 44 studies stated that the accuracy of endoscopic biopsies, EUS, and ^18^F-FDG PET(/CT) as single modalities for detecting residual disease after neoadjuvant chemoradiotherapy for esophageal cancer is insufficient [63]. A recommendation not to use ^18^F-FDG PET to guide post-RCT decisions in patients with esophageal cancer was already made in 2010 on the basis of 20 reports [64], and the same recommendation is, for example, still included in the current German S3 guideline for esophageal cancer [55]. The reported ranges for cCR prediction accuracy and negative predictive values (NPV) (56–88%, 35–94%) of ^18^F-FDG PET/CT [59] leave room for improvement of the corresponding PET/CT diagnostics by using more tumor-specific tracers.

### Tracer development and translation

Comparison of preclinical data for ^68^Ga-D0103 and ^68^Ga-Trivehexin (see Fig. 3 and Fig. 5) suggested a generally lower background and less nonspecific uptake of ^68^Ga-D0103. Our first clinical ^68^Ga-D0103 PET scan did not provide clear evidence for such an advantage, which highlights a limited correlation of preclinical studies in rodents with human data. However, given the large interindividual deviations of the general biodistribution of ^68^Ga-Trivehexin [41], the hypothesis can neither be confirmed nor refuted on the basis of a series of ^68^Ga-D0103 PET scans for a single patient, assuming that biodistribution of this tracer is also variable. Tumor uptake of ^68^Ga-D0103 after 104 min was higher than at the earlier time points (15, 45, and 90 min p.i.), which suggests that uptake of ^68^Ga-D0103 apparently progresses over an even longer period of time. In accordance with preclinical biodistribution data, ^68^Ga-D0103 showed a longer tumor retention in humans than ^68^Ga-Trivehexin, which reached its maximum tumor uptake in pancreatic ductal adenocarcinoma (PDAC) already at approximately 20 min p.i. [41] and slightly decreased average tumor uptakes at 120 min p.i. [40]. The absorbed effective dose for ^68^Ga-D0103 is very likely within the range known for ^68^Ga-labeled radiotracers, especially since the variation is small for dose coefficients of radionuclides with relatively short half-lives [65]. For example, assuming the dose coefficient for ^68^Ga-HA-DOTATATE of 0.0257 mSv/MBq [66], the whole-body PET with the activity administered here would result in an effective dose of approximately 4.9 mSv, which is very similar to the effective dose calculated for ^68^Ga-Trivehexin (4.7 mSv). For a more accurate estimation of the effective dose, organ-specific tracer kinetics would be necessary at more time points. An estimate of the organ-specific tracer kinetic based on the data obtained here can be found in the Supplemental Information.

In view of the high uptake of ^68^Ga-D0103 in the primary tumor and especially in a lymph node metastasis at later time points, the present clinical case suggests a potential added value of ^68^Ga-D0103 PET/CT for imaging of lymphatically metastasized ESCC in a challenging setting. This finding fully met our expectations as our IHC analysis showed that 99% of the immunohistochemically investigated human ESCC cases displayed membranous αvβ6-integrin positivity for tumor cells, and furthermore 48% of those exhibited a strong membranous expression for αvβ6-integrin (see Fig. 2). Of note, previously reported clinical data already suggested an insignificant uptake of αvβ6-integrin PET tracers in cancer-associated inflammation because αvβ6-integrin is not specifically upregulated in inflamed areas [40]. Therefore, it seems worthwhile to further evaluate the ESCC specificity of ^68^Ga-D0103 PET in comparison to ^18^F-FDG PET, and to assess the clinical value of αvβ6-integrin PET/CT for ESCC diagnostics at different stages of the patient journey in prospective clinical trials. As ITGB6 expression in ESCC was not dependent from tumor grade, it is expected that ^68^Ga-D0103 PET/CT should perform equally well for patients at any stage.

This work has several limitations. Since the preclinical models are based on immune-compromised animals, the αvβ6-integrin mediated TGF-β activation and its interplay with the immune system might not be reflected. Furthermore, the clinical evaluation based on a single patient restricts the generalizability of ^68^Ga-D0103 PET/CT's diagnostic performance and reproducibility. Larger clinical cohorts will be required to validate diagnostic accuracy, sensitivity, and specificity, particularly for restaging and metastasis detection. In addition, a rigorous correlation of PET uptake with αvβ6-integrin expression levels (IHC) is needed to establish the link between αvβ6-integrin abundance, PET data, and clinical outcomes (e.g., survival, recurrence, or response to therapy), and to define prognostic and therapeutic implications.

## Conclusion

The nearly complete (99%) αvβ6-integrin positivity of ESCC and the high average expression density on ESCC tumor cell membranes indicate a high potential of this receptor as a theranostic target in this cancer entity. Our study indicated a potential value of αvβ6-integrin PET/CT imaging for re-staging and therapy guidance in certain cases. The prolonged tumor retention of ^68^Ga-D0103 furthermore suggests a therapeutic potential of αvβ6-integrin targeted radiopharmaceuticals when labeled with radionuclides like ^177^Lu, ^161^Tb, or ^225^Ac. Further studies on αvβ6-integrin targeted theranostics of ESCC are thus warranted.

## Supplementary Information

Below is the link to the electronic supplementary material.

Supplementary file1 (PDF 33596 KB)

Supplementary file2 (XLSX 39 KB)

## Data Availability

The datasets used and/or analyzed during the current study are available from the corresponding author on reasonable request.
