# Supplementary material for: PET/CT imaging of esophageal cancer targeting tumor cell specific αvβ6-integrin expression
Source: Eur J Nucl Med Mol Imaging. 2025 Jun 20;53(1):607–18. doi: 10.1007/s00259-025-07408-7 (PMC12660471; doi:10.1007/s00259-025-07408-7)
Supplement: Supplementary file 1 — Supplementary file1 (PDF 33596 KB) [file 259_2025_7408_MOESM1_ESM.pdf]

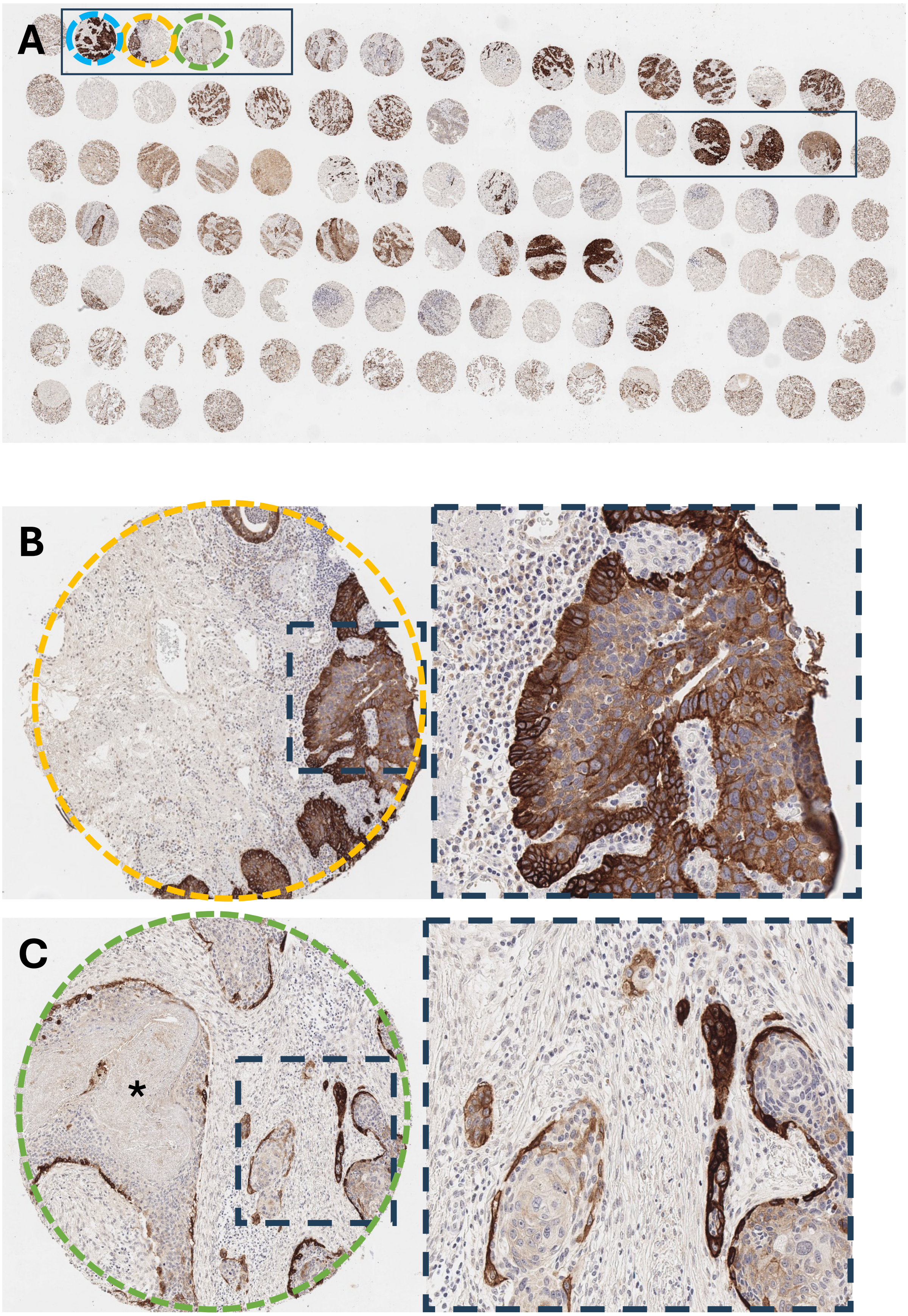
A
B
C
*

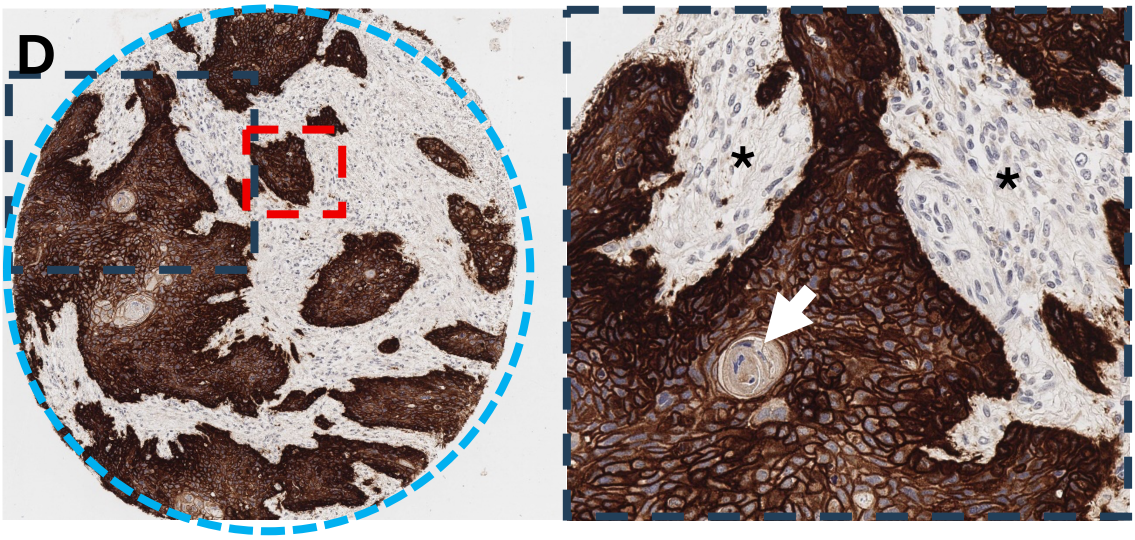

Figure S1. Case example for a final score of 3. This patient diagnostic case was represented by in total 8 scored tissue cores (each 1.1 mm in diameter) on the generated and B6ITG-IHC stained tissue micro array (**A**). The black boxes frame the cores of the same patient case. Three exemplary cores are shown in B-D as an overview (8 ×) and close-up (20 ×).

A Tissue micro array (TMA). The variability in staining intensities can be estimated from subgross magnification. Please note that the TMA is framed by placental tissue serving as a wall. **B** The majority of tumor cells in this core display a high (score 3) membranous ITGB6 expression. **C** Few tumor cells display a high membranous ITGB6 expression, some stain less intense and some tumor cells as well as keratinization (*) are negative for ITGB6. **D** All tumor cells display a high membranous ITGB6 expression. Keratin pearl produced by the tumor is negative for ITGB6 (arrow) as is the tumor stroma (*). The red box corresponds to Figure 1.

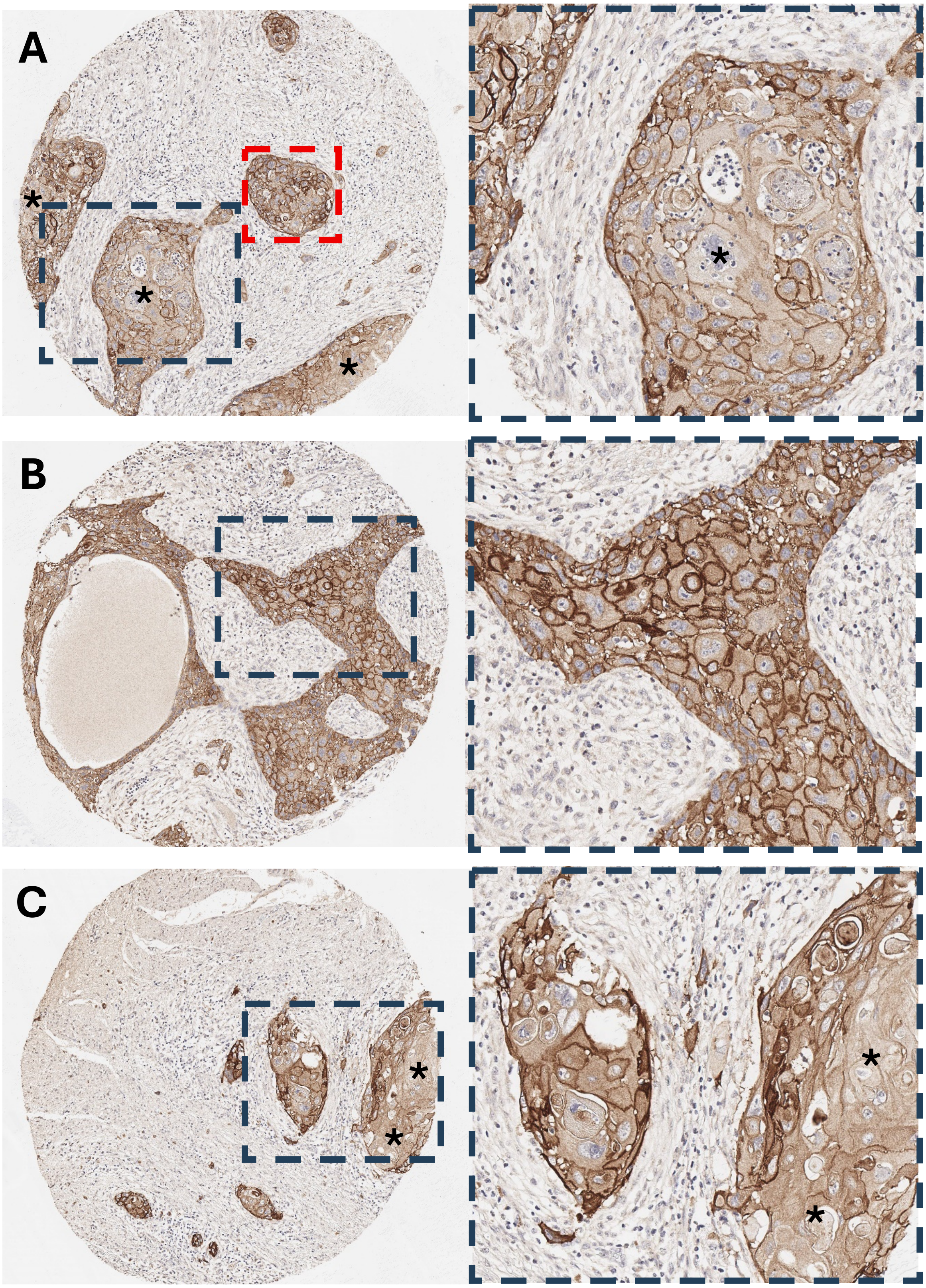
A
B
C

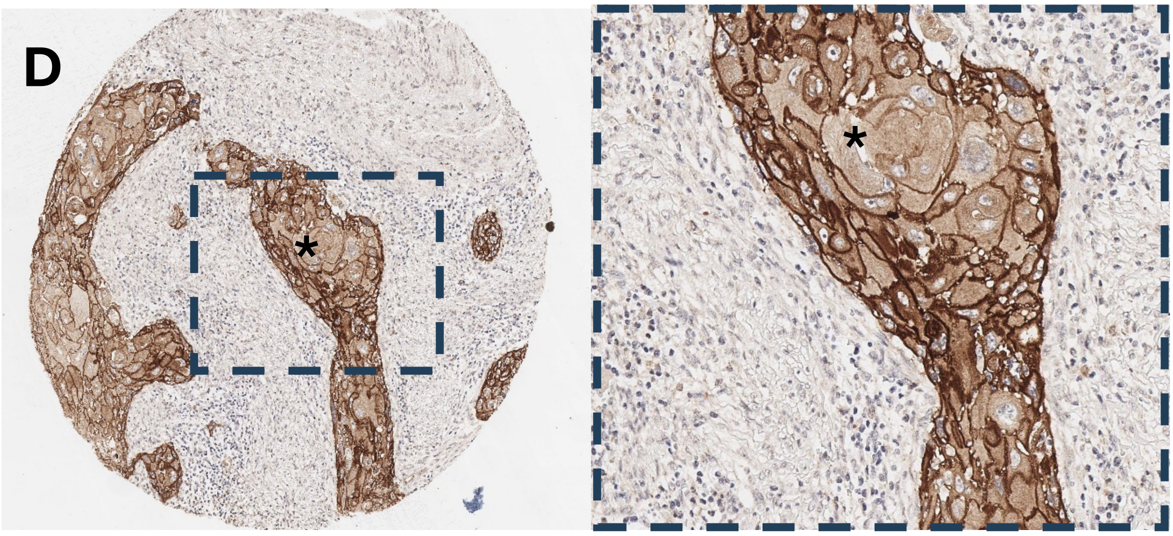

Figure S2. Case example for final score of 2 that was shown in Figure 2. This patient diagnostic case was represented by in total 4 tissue cores (each tissue core approximately 1.1 mm in diameter) on the generated and IHC stained tissue micro array. **A** The majority of 80 % of tumor cells included in this specific tissue core show moderate (score of 2) membranous ITGB6 expression. **B** 100 % of tumor cells in this specific tissue core show moderate membranous ITGB6 expression. **C** 90 % of tumor cells in this specific tissue core show moderate ITGB6 expression. **D** 90 % of tumour cells in this specific tissue core show moderate ITGB6 expression. Note the cytoplasmic signal in all tumor cells, which was not taken into account for scoring. * = tumor cells with an expression of ITGB6 < 2.

The final score was calculated as follows: (2×0.8 + 2×1 + 2×0.9 + 2×0.9)/4 = 1.8

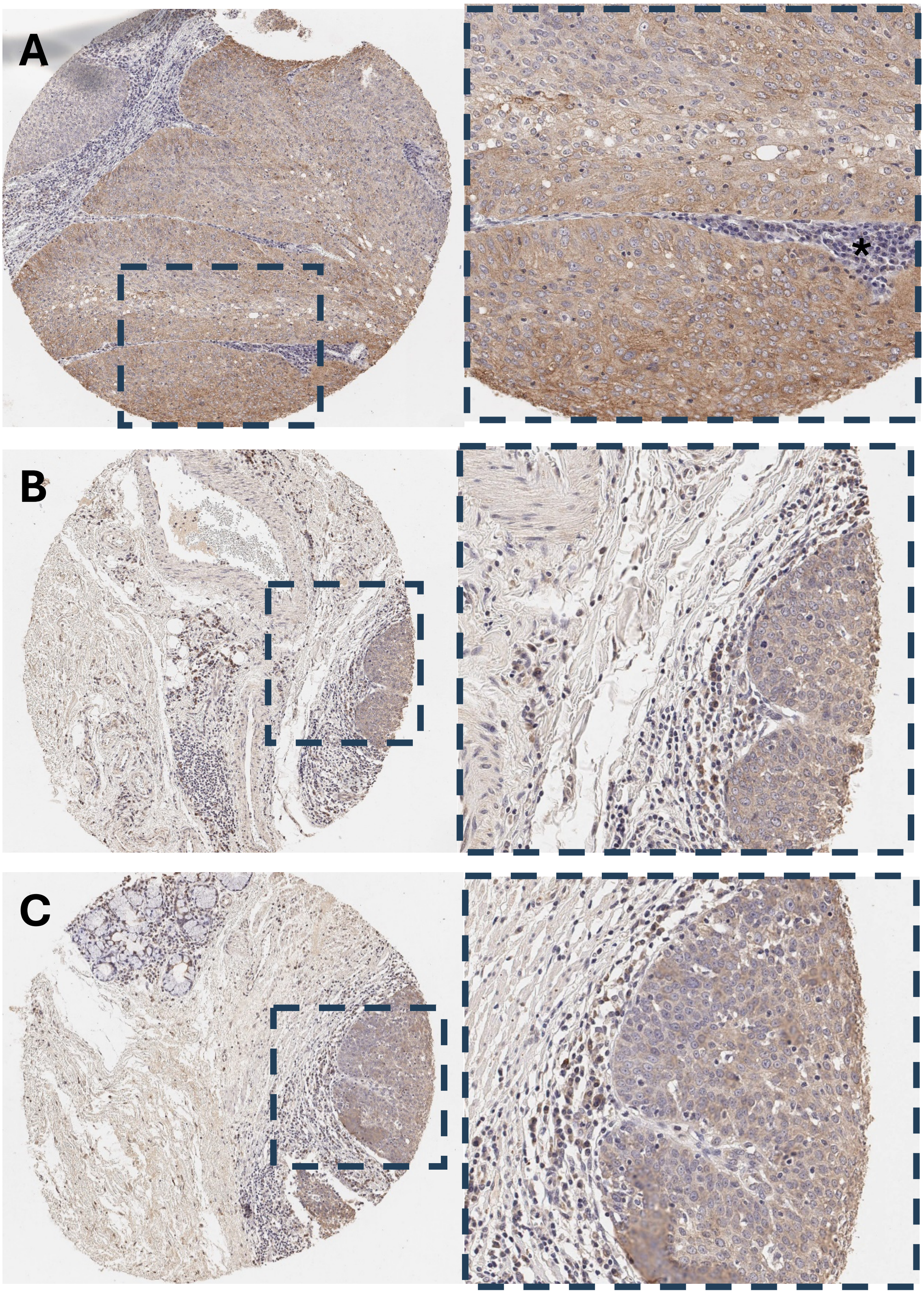

Figure S3. Case example for final score of 1. This patient diagnostic case was represented by in total 3 tissue cores. **A** On one tissue core some tumor cells displayed very mild membranous positivity for ITGB6 (score 1), especially in the lower part of the core. Infiltrating immune cells (*) are ITGB6-negative; however, only tumor cells were evaluated for scoring (*). **B, C** Tumor cells on these other cores are completely negative for membranous ITGB6, however, mild cytoplasmic positivity is evident.

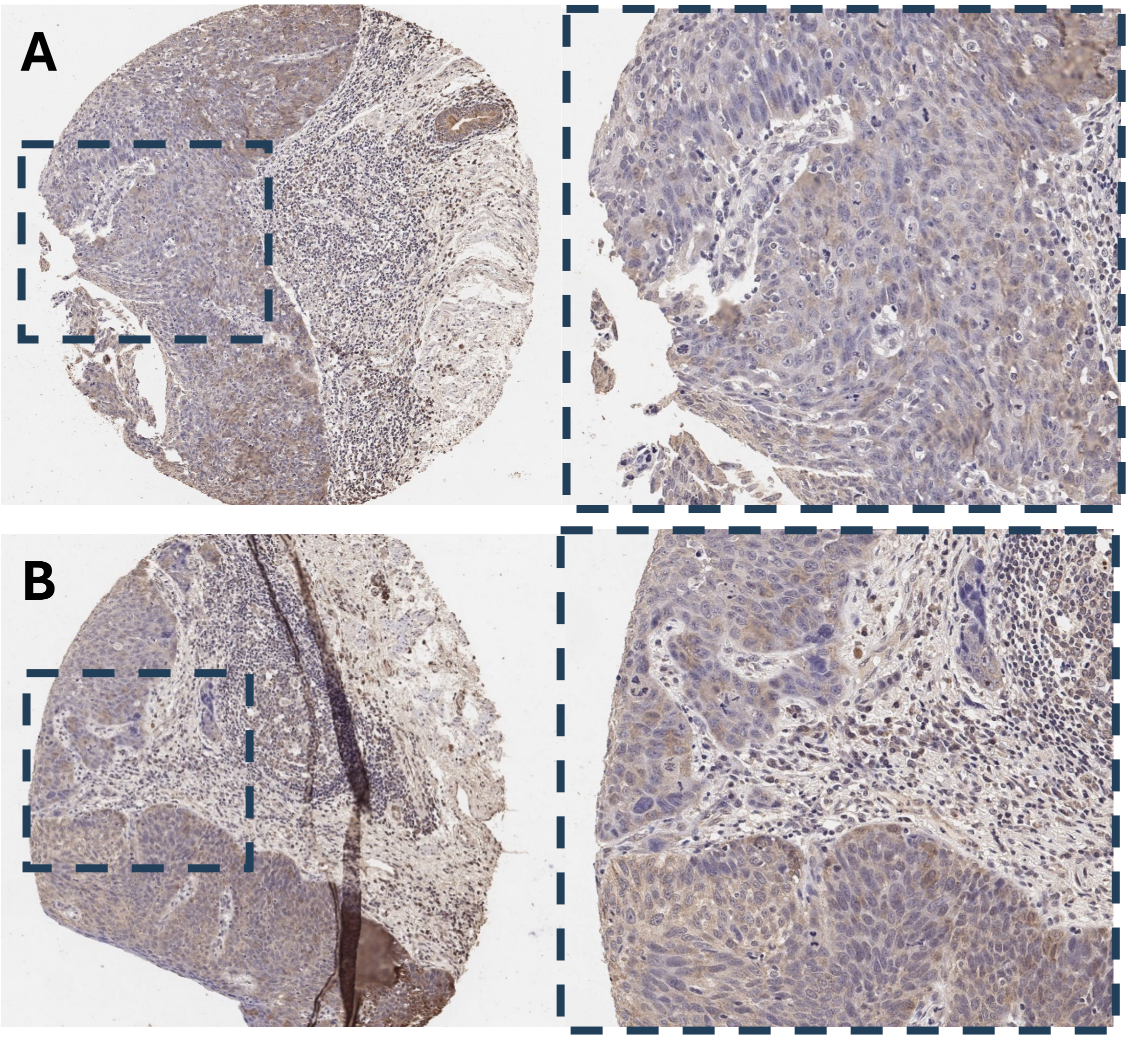

Figure S4. Case example for final score of 0. A, B All tumor cells are negative for membranous ITGB6; however, diffusely mild to moderate cytoplasmic positivity is evident.
